# Epigenetic reprogramming in the porcine germ line

**DOI:** 10.1186/1471-213X-11-11

**Published:** 2011-02-25

**Authors:** Sara MW Hyldig, Nicola Croxall, David A Contreras, Preben D Thomsen, Ramiro Alberio

**Affiliations:** 1 Department of Basic Animal and Veterinary Sciences, Faculty of Life Sciences, University of Copenhagen, 1870 Frederiksberg C, Denmark; 2 Division of Animal Sciences, School of Biosciences, University of Nottingham, Loughborough, LE12 5RD, UK

## Abstract

**Background:**

Epigenetic reprogramming is critical for genome regulation during germ line development. Genome-wide demethylation in mouse primordial germ cells (PGC) is a unique reprogramming event essential for erasing epigenetic memory and preventing the transmission of epimutations to the next generation. In addition to DNA demethylation, PGC are subject to a major reprogramming of histone marks, and many of these changes are concurrent with a cell cycle arrest in the G2 phase. There is limited information on how well conserved these events are in mammals. Here we report on the dynamic reprogramming of DNA methylation at CpGs of imprinted loci and DNA repeats, and the global changes in H3K27me3 and H3K9me2 in the developing germ line of the domestic pig.

**Results:**

Our results show loss of DNA methylation in PGC colonizing the genital ridges. Analysis of *IGF2-H19 *regulatory region showed a gradual demethylation between E22-E42. In contrast, DMR2 of *IGF2R *was already demethylated in male PGC by E22. In females, *IGF2R *demethylation was delayed until E29-31, and was de novo methylated by E42. DNA repeats were gradually demethylated from E25 to E29-31, and became de novo methylated by E42. Analysis of histone marks showed strong H3K27me3 staining in migratory PGC between E15 and E21. In contrast, H3K9me2 signal was low in PGC by E15 and completely erased by E21. Cell cycle analysis of gonadal PGC (E22-31) showed a typical pattern of cycling cells, however, migrating PGC (E17) showed an increased proportion of cells in G2.

**Conclusions:**

Our study demonstrates that epigenetic reprogramming occurs in pig migratory and gonadal PGC, and establishes the window of time for the occurrence of these events. Reprogramming of histone H3K9me2 and H3K27me3 detected between E15-E21 precedes the dynamic DNA demethylation at imprinted loci and DNA repeats between E22-E42. Our findings demonstrate that major epigenetic reprogramming in the pig germ line follows the overall dynamics shown in mice, suggesting that epigenetic reprogramming of germ cells is conserved in mammals. A better understanding of the sequential reprogramming of PGC in the pig will facilitate the derivation of embryonic germ cells in this species.

## Background

Primordial germ cells derived from the epiblast of pre-gastrulating embryos are the founder population of the future gametes. A unique attribute of PGC is the acquisition of totipotency, which is required for the generation of a new organism. Extensive epigenetic reprogramming of PGC underlies the capacity of these cells for acquiring totipotency [1,2]. Genome-wide DNA demethylation in mouse PGC results in the complete erasure of methylation marks in single-copy and imprinted genes, and a moderate reduction in retrotransposons and other repetitive elements [3-5]. This demethylation is a unique reprogramming event, most of which is restricted to a short window of time between E10.5-13.5 in the mouse, and is critical for erasing epigenetic memory and preventing the transmission of epimutations to the next generation [3,4,6]. Just before these major DNA demethylation events, changes in histone marks contribute to the establishment of a distinctive chromatin signature in PGC [1]. Reduction in H3K9me2 is followed by an increase in H3K27me3 levels in migrating mouse PGC between E7.75 and E8.75, at a time when these cells undergo G2 arrest and transcriptional quiescence [3,7]. When the PGC reach the genital ridges they undergo major conformational changes including loss of linker histone H1 and replacement of nucleosomal histones [8]. Together, these dynamic events define a critical period for the epigenetic reprogramming of the mouse germ line.

Most of our knowledge in mammalian germ line development originates from studies in mice. A recent study demonstrated that mouse and rat embryonic germ (EG) cells share common ground state properties, suggesting that the molecular circuitry of pluripotency is conserved in rodents [9]. Very little is known about the sequence of events during PGC development in other species [10], and studying these events in non-rodents is important for establishing the conserved mechanisms of PGC development in mammals.

The pig is a good model for studying mammalian development, due to the developmental and physiological similarities with most other mammals, including humans. Furthermore, the pig is also excellent for modelling human disease, and therefore great effort has been devoted to develop efficient genetic modification technologies in this species [11]. Pig EG cell lines derived from gonadal PGC of E28-35 embryos have been used to generate transgenic animals [12]. In the pig, migratory PGC can be identified in the dorsal mesentery of the hindgut in E18-20 and the colonisation of the genital ridges occurs around E23-24 [13]. However, the events characterizing the epigenetic reprogramming of pig PGC remain largely unexplored. A recent report showed demethylation of the differentially methylated domain of *IGF2-H19 *gene cluster and centromeric repeats between E24-E28 followed by de novo methylation in male PGC by E30-E31, demonstrating that major DNA demethylation occurs in the pig germ line shortly after colonizing the gonadal ridges [14]. There is also evidence that the imprinted gene *PEG10 *is biallelically expressed in EG cells derived from E27 embryos, indicating that demethylation has occurred [15]. In the present study we extended these initial observations by investigating the methylation reprogramming of imprinted genes, retrotransposons and genome-wide histone modifications in migratory and gonadal PGC. We show that imprinted gene demethylation occurs asynchronously in pig PGC, with *IGF2-H19 *demethylation not beginning before E22, and *IGF2R *demethylation already starting in male PGC at this time point. We also show that SINE repeats undergo moderate progressive demethylation between E22-E31. Finally, we show that migratory pig PGC undergo reprogramming of H3K27me3 and H3K9me2 concurrent with a G2 arrest.

## Results & Discussion

### OCT4 expression identifies the early pig germ line

**Figure 1:**
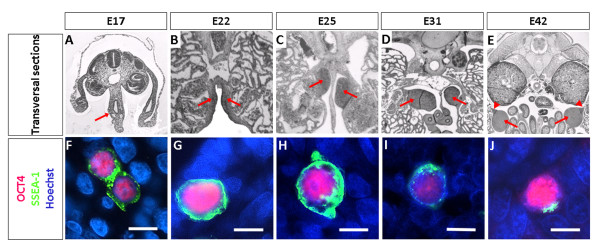
**Identification of PGC by immunostaining**. The top panel shows transversal sections of porcine embryos in the area where the PGC are found; hind gut of E17 (Figure 1A), genital ridges or primitive gonads of E22 (Figure 1B), E25 (Figure 1C), E31 (Figure 1D) and E42 (Figure 1E). Arrows indicate the PGC containing tissue (hind gut, genital ridges or gonads). Arrowhead depicts the mesonephric connection. The bottom panel shows double fluorescence immunostaining of the OCT4 and SSEA-1 in transversal sections of porcine embryos of the ages E17 (Figure 1F), E22 (Figure 1G), E25 (Figure 1H), E31 (Figure 1I) and E42 (Figure 1J). 5-7 PGC containing sections of one embryo of each stage were stained. Scale bars = 10 µm

In mice, *Oct4 *(also known as *Oct3/4 *and *Pou5f1*) plays a critical role during the specification of PGC precursors [16] and is required for germ cell survival in late migratory stages [17]. Cell type specific expression of this marker has been demonstrated in migratory PGC [18] and can be used to isolate these cells using fluorescence activated cell sorting (FACS) [8]. During pig development, OCT4 expression is detected in the pluripotent epiblast and becomes confined to migratory PGC by E17 [19]. To determine the suitability of OCT4 for identifying pig germ cells in late migratory and early gonadal stages we performed antibody based staining of OCT4 in combination with SSEA-1, another known germ line marker [13], in sections of embryos between E17 and E42 (Figure 1). In E17 embryos, OCT4/SSEA1 cells were identified mostly in the hindgut with a few of them approaching the position of the genital ridges, which has not yet formed (Figure 1A). The PGC are large spherical cells with strong specific OCT4 nuclear localisation and SSEA-1 staining of the plasma membrane (Figure 1F). In E22 PGC, which are positioned in the primordium of the genital ridges, we detected clear OCT4/SSEA1 staining (Figure 1B,G). The genital ridges begin to take the shape of early gonads at E25, a process that continues in E31 embryos (Figure 1C,D). PGC maintain OCT4/SSEA-1 staining in E25 PGC, however, SSEA-1 specific staining was noticeably weaker in many of the OCT4 positive cells in E31 PGC (Figure 1H,I). By E42 the tissue of the early gonads has begun organising. At this age, germ cell cords are present in both male and female gonads, though larger and more regular in males. Male gonads are rounded with only a slim cellular connection to the mesonephros [20]. The specimen shown here fulfilled the criteria of male gonad with a characteristic attenuated appearance of the mesonephric connection and well defined large cords (Figure 1E; Additional File 1). The expression of the two markers was somewhat inconsistent and several putative germ cells expressed only one of the two markers (not shown). Most cells, however, still expressed both (Figure 1J). Down regulation of Oct4 is seen in the mouse female germ line around E17.5 coinciding with the time of entry into meiosis. In males, Oct4 expression, however, does not decrease [18]. In our study we see down regulation of this marker in some individual male germ cells (data not shown).

These results show that OCT4 is expressed in migratory and early gonadal PGC and can be used as a reliable marker of pig PGC between E17-E31. Furthermore, it is expressed in the majority of putative germ cells at E42. We therefore used OCT4 staining followed by FACS sorting to obtain purified PGC at different developmental stages.

### Reprogramming of gender specific methylation imprints at CTCF3 in *IGF2-H19 *gene cluster is initiated after germ cell arrival to the genital ridges

**Figure 2:**
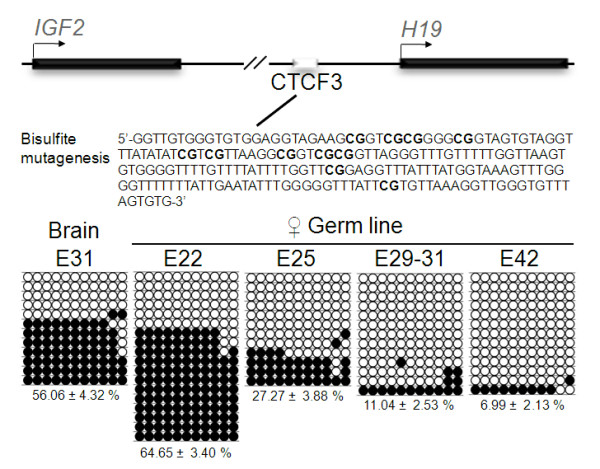
**Methylation dynamics of the *IGF2-H19 *gene cluster**. Methylation of the CpG regulatory box CTCF3 region for *IGF2-H19 *gene cluster was investigated by bisulfite sequencing. A DNA pool from germ cells of 6-8 embryos of each gender in the stages E22, E25, E29-31 and E42 was bisulphite converted and used for the analysis after one PCR reaction and subsequent transformation and cloning. The position of the CTCF3 is indicated on the schematic representation of the gene cluster and the sequence of the investigated fragment after bisulfite mutagenesis is showed below. Empty and filled circles indicate unmethylated and methylated CpGs, respectively. 12-18 clones were analysed from each group. Each horizontal line represents one clone. Percent methylation mean ± SEM for each group is indicated below.

Demethylation of imprinted genes occurs in PGC located in the genital ridges between E10.5-E13.5 in mice [4], and this event appears to progress synchronously for most imprinted genes [21] including the *Igf2-H19 *gene cluster [22]. To determine the timing of demethylation of the paternally imprinted control region of the pig *IGF2-H19 *gene cluster we examined this region after bisulfite conversion of DNA extracted from purified PGC. Our analysis focussed on one of the binding sites for the insulator protein CTCF, since this site has previously been shown to be differentially methylated in somatic tissues and reprogrammed prior to E24 during porcine germ line development [14]. We determined that the level of methylation in PGC at the time of arrival to the genital ridges (E22) was not below the 50% expected for a monoallelic methylated sequence, indicating that DNA demethylation has not initiated at this stage in male and female PGC (Figure 2 and data not shown). Samples from pig brain showed a typical pattern of differential methylation for this region (56.06%), as expected for somatic cells. In contrast, DNA methylation decreased significantly in female PGC at E25 (27.27%) and was followed by further reduction at E29-31 (11.04%) and E42 (6.99%). The results indicate that demethylation of CTCF3 begins in PGC shortly after they arrive to the genital ridges and de novo methylation is not resumed in female PGC at E42. This pattern of imprint demethylation follows closely the dynamic reported in the mouse differentially methylated domain of *Igf2-H19 *[23]. Re-establishment of imprints occurs in mouse male germ cells from E14.5 starting with the paternal allele [24]. De novo methylation of this region occurs by E31 in male pig PGC [14]. Lack of polymorphism information restricted our capacity to establish the dynamic of paternal allele methylation, as established in mice. However, the evidence that i) CTCF3 is fully methylated in pig sperm and unmethylated in oocytes [14], ii) biparental embryos show almost complete demethylation in female PGC between E25 and E42, and iii) de novo methylation occurs in male PGC, supports the idea that the paternal allele is subject to methylation reprogramming in the pig.

### Reprogramming of gender specific imprints of the *IGF2R *gene is initiated in porcine germ cells prior to arrival in the genital ridges

**Figure 3:**
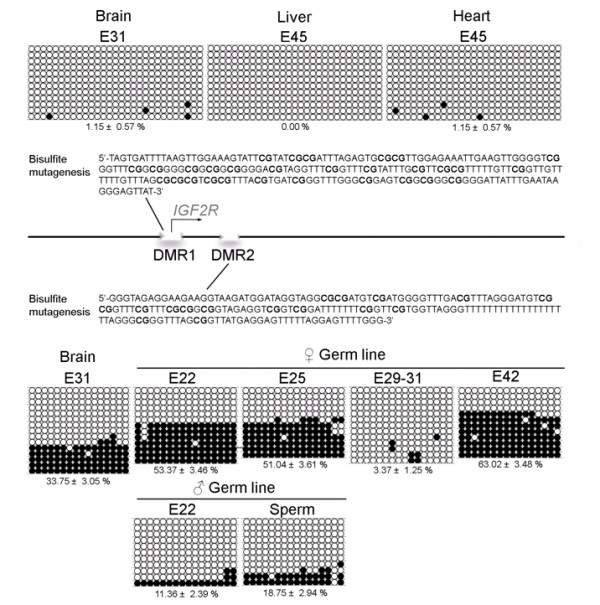
**Methylation dynamics of the *IGF2R *gene**. The two CpG islands of the *IGF2R *gene are known from other species as Differentially Methylated Region 1 (DMR1) and 2 (DMR2). A fragment of these regions was investigated for methylation of CpGs (See Additional file 2). The positions of the DMRs are indicated on the schematic representation of the gene and the sequences of the investigated fragments after bisulfite mutagenesis are showed above and below, respectively. DNA from liver and heart of an E45 embryo and a pool of DNA from ten E31 brains were analysed for DMR1. A DNA pool from germ cells of six-eight embryos of each gender in the stages E22, E25, E29-31 and E42 was used for the analysis of DMR2. Furthermore, DNA pools from a sperm sample and from ten E31 brains were included. The DNA was bisulphite converted and used for the analysis after one PCR reaction and subsequent transformation and cloning. Empty and filled circles indicate unmethylated and methylated CpGs, respectively. 11-15 clones were analysed from each group. Each horizontal line represents one clone. Percent methylation mean ± SEM for each group is indicated below.

The *IGF2R *gene is imprinted in rodents, artiodactyls and marsupials, but is biallelically expressed in primates [25,26]. Imprinting regulation in the mouse *Igf2r *depends on two differentially methylated regions (DMRs): DMR1 located in the promoter region and DMR2 in intron 2 (DMR2), representing the primary imprinting signal for this gene [27,28]. Although it has been shown that *IGF2R *is imprinted in the pig [26,29], there is no information on the imprinting control region for this gene. We performed this analysis from the recently published pig genome sequence. The putative porcine *IGF2R *gene is located on chromosome 1 between 8.50 Mb and 8.60 Mb (Additional File 2). The gene structure is very similar to orthologues from other species such as human, mouse and cow, but alignments showed that while the mRNA sequences are highly homologous, the intron sequences demonstrate low conservation between species (data not shown). We confirmed that the porcine promoter region contains a CpG island spanning the entire predicted exon 1 as seen in other described mammalian *IGF2R *genes [30,31]. Furthermore, we identified the large CpG island of intron 2, also present in human, mouse, dog, sheep, and cow, but absent in chicken, lemur, tree shrew, opossum or platypus [26,31]. Additional File 2B shows CpG distribution in the two predicted CpG islands. In the mouse, the CpG island in the promoter region of the *Igf2r *is methylated in the repressed paternal allele, but unmethylated in the active maternal allele. This DMR is unmethylated in both alleles in opossum and domestic dog despite the imprinted status of the gene [31,32]. Here we examined the methylation status of the porcine DMR1 in fetal brain by direct sequencing of bisulfite converted DNA and found no CpGs methylation (data not shown). We confirmed these findings by sequencing individual clones from brain, heart and liver DNA (n = 12, 12 and 12 respectively), which show almost complete demethylation (Figure 3). To exclude the possibility of PCR bias favouring unmethylated DNA we methylated genomic DNA using Sss1 prior to bisulfite conversion. A PCR fragment was obtained from the methylated sample (not shown), indicating that our observations with unmethylated DNA are not due to PCR bias. These results indicate that DMR1 in the pig *IGF2R *is not differentially methylated.

We next examined the methylation status of the DMR2 located in intron 2, which is maternally methylated in mice [33], human [34], cattle [35] and sheep [36]. Our analysis from bisulfite converted brain DNA showed that this region is differentially methylated (Figure 3), suggesting that this region plays a role in imprinting control of the pig *IGF2R*. We used this fragment to investigate the dynamic methylation reprogramming in purified PGC from porcine embryos of different developmental stages. In mice, DMR2 demethylation of *Igf2r *begins as early as E9.5 in migratory PGC [37], indicating that a gonadal environment is not needed to initiate DNA demethylation. We found that only male porcine PGC from E22 embryos show low levels of methylation with only 11.36% methylated CpGs. Gender specific differences were not observed in the methylation level of this gene in migratory mouse PGC [37]. Importantly, although at this developmental stage the gonadal primordium has the characteristics of an indifferent gonad [38], *SRY *and its downstream target *SOX9 *are expressed in the migratory path of pig PGC between E21-E23 [39,40], indicating that at the molecular level sexual dimorphism has already been established. Thus, demethylation of *IGF2R *in male PGC provides evidence supporting sex specific differences in the germ cells at this stage. The levels of methylation remained low in mature pig sperm (Figure 3), in agreement with *Igf2r *methylation reported in mice [41] and sheep sperm [42].

Interestingly, early gonadal PGC from female E22 and E25 embryos showed approximately 50% methylation, indicating that demethylation had not yet initiated. In PGC from female E29-31 embryos this DMR2 was almost completely demethylated, and by E42 the methylation level reached 63%, indicating de novo methylation by this stage (Figure 3). Since the same E42 samples were used to analyse the methylation status of *H19*, which is almost completely unmethylated in PGC (Figure 2), we think it is unlikely that the samples were contaminated with somatic cells. In mice the *Igf2r *DMR2 remains unmethylated in female germ cells until after birth, where de novo DNA methylation is acquired during oocyte growth [43,44]. The precocious de novo methylation observed in female pig PGC suggests that acquisition of DNA methylation in the *Igf2r *is controlled differently in the two species. In line with our observations, a recent report showed that sheep oocytes derived from small preantral follicles possess a monoallelic pattern of methylation [42], indicating that precocious *IGF2R *methylation also occurs in sheep.

Together, our results demonstrate that imprinted DMR2 of *IGF2R *in the pig undergoes methylation reprogramming, with a precocious onset of demethylation in male migratory PGC, and early de novo methylation initiated in female germ cells before birth.

### Short Interspersed Nuclear Elements are partially demethylated in the developing germ line

**Figure 4:**
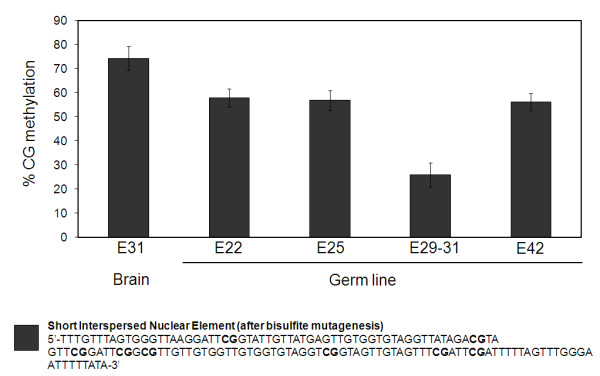
**Methylation dynamics of short interspersed repeats**. Short Interspersed Nuclear Elements (SINE) were investigated for their methylation level in the porcine germ line. A DNA pool from germ cells of 13-16 embryos of the stages E22, E25, E29-31 and E42 was bisulphite converted and used for the analysis after one PCR reaction and subsequent transformation and cloning. 11-24 clones were analysed from each group. Due to high mutagenic rate in this type of elements, single clones are not identical regarding number and position of CpGs. The mean methylation level was calculated as suggested by Yang et al. [52] and results shown in the diagram. The sequence of an example of the investigated fragments after bisulfite mutagenesis is shown. Bars on the columns indicate SEM. E: embryonic stage.

Retrotransposable elements are abundant repeat sequences in the genome subject to methylation reprogramming during early embryo development [45] and in mouse PGC arriving to the primitive gonad [4,46]. In the porcine genome, they are diffusely distributed in the euchromatic chromosomal regions, i.e. away from centromeric DNA repeat blocks [47]. Demethylation of repeats, such as SINE, occurs during pig preimplantation development [48], however there is only limited information on how these repeats are reprogrammed in PGC. Analysis of centromeric DNA repeats shows that these sequences are demethylated extensively between E26-E31 in female PGC, however male PGC show only moderate demethylation by E28 and are remethylated by E31 [14]. We investigated the methylation dynamics of SINE repeats after bisulfite sequencing analysis of DNA obtained from PGC. Because of the high polymorphism within repeat sequences, individual clones did not have identical numbers of CpGs. Thus, the total methylation level for each examined group was calculated. The methylation level was investigated in gender separated DNA, but since we found no differences between genders, the data presented represents the collective data (Figure 4). SINE repeats were highly methylated in control DNA from brain of E31 embryos (74.4%). In PGC we detected lower levels of methylation in E22 (58.0%) and E25 (56.8%), reaching the lowest level E29-31 (26%). This was followed by an increase at E42 (56.1%), indicating that de novo methylation had resumed by this time. The dynamic demethylation observed in our experiments are in agreement with the overall pattern of DNA demethylation observed for LINE1, SINE and other repeats such as IAPs in mouse gonadal PGC between E11.5-E13.5 [4,5,46]. However, the interval needed for demethylation of repeats in the pig appears to be extended over a period of 8-10 days from around E22-E31.

The overall reduction in methylation of SINE repeats is lower compared to the reported demethylation of centromeric repeats, which show extensive and gender specific demethylation in PGC at similar stages [14]. This suggests that the different genomic contexts of interspersed versus centromeric repeats can impact on the demethylation machinery in PGC.

### Cell cycle distribution and dynamics of histone modifications in porcine PGC

**Figure 5:**
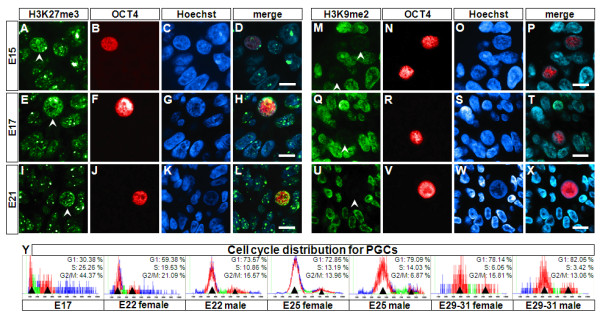
**Cell cycle distribution and H3K27 trimethylation and H3K9 dimethylation in porcine PGC**. Reprogramming of histone modifications H3K27me3 and H3K9me2 was investigated by immunohistochemistry in paraffin sections of porcine E15 (n = 1, Figure 5A-D, M-P), E17 (n = 1, Figure 5E-H, Q-T) and E21 (n = 1, Figure 5I-L, U-X) embryos. Micrographs show the histone modifications in green (Figure 5A, E, I, M, Q, U). PGC are identified by OCT4 expression in red and counterstained with Hoechst for DNA stain in blue. Arrowheads mark PGC. Figure 5Y shows the cell cycle distribution after FACS analysis of PGC during development (n = 13-24 for each stage). Arrowheads denote the G1 and G2 peaks. Scale bars = 10 μm.

Epigenetic reprogramming in the mouse germline includes changes in histone modifications occurring before the cells arrive to their definitive location in the gonadal ridges [1,2]. During mouse PGC migration through the hindgut a progressive loss of di-methylation of lysine 9 on histone 3 (H3K9me2) takes place, reaching almost complete erasure by E8.75 [7]. The reduction in H3K9me2 precedes the increase in the levels of the repressive tri-methylation of lysine 27 on histone 3 (H3K37me3) mark, which is established from E8.25 and maintained in PGC until E10.5 [7,8]. The changes in histone modifications occur in PGC arrested in G2 of the cell cycle, defining a clear window of time for epigenetic reprogramming [2]. There is currently no information on the similarities in epigenetic reprogramming of the germ cells in other mammals. We therefore investigated whether these histone marks are reprogrammed in migratory pig PGC between E15-E21 (Figure 5A-X). We found that H3K27me3 was higher in PGC migrating through the hindgut of E15 embryos than their somatic neighbours (Figure 5A-D), and this mark remained high in E17 and E21 (Figure 5E-L). By contrast, H3K9me2 staining was reduced in PGC compared to their somatic neighbours in E15 (Figure 5M-P) and in E17 (Figure 5Q-T), and was completely erased from PGC in E21 (Figure 5U-X). We find that acquisition of H3K27me3 occurred before H3K9me2 was completely erased, suggesting that the extended window of time required for histone remodelling in the pig allows for a continuum in the sequence of events.

Next, we examined the DNA content of FACS sorted PGC to determine their cell cycle stage. The earliest stage of PGC that we were able to isolate was from E17 embryos, which showed a great proportion of cells in G2 (44%). This distribution resembles the patterns reported for murine PGC at about E9.75, a time point just following the G2 arrest observed between E7.5-E9 in the PGC population [7]. In contrast, the porcine PGC from E22, E25 and E29-31 show nearly identical distribution displaying a clear G1 peak, a small broad S phase and a minor G2 peak (15-21%) (Figure 5Y). This cell cycle distribution resembles that of mouse somatic cells [49], and that of the somatic fraction of the porcine cell suspension used for sorting in this study (data not shown). These results show that the dynamic changes in H3K27me3 and H3K9me2 in pig PGC correspond overall with the pattern described for mouse migratory PGC [3]. It is interesting however, that we observe these dynamic changes occurring over a longer period of about 6 days, which is more than three times the interval required in mice. The protraction of this process is likely due to the slower development in the pig.

## Conclusions

**Figure 6:**
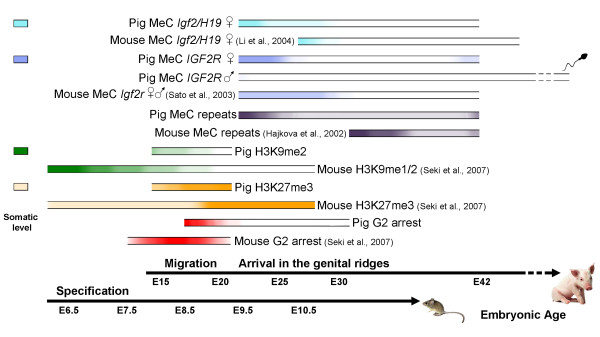
**Diagramatic representation of the dynamic events during reprogramming of the germ cells in the mouse and the pig**. Schematic overview of the events studied in the current report compared with the same events in mouse PGC. Erasure of *Igf2/H19 *imprints occurs in gonadal PGC of both species. Male pig migratory PGC lose *IGF2R *imprints before reaching the gonads, in contrast to the findings in mice [37], where demethylation occurs at the same time in male and female PGC after entering the gonad. Remodeling of repetitive sequences follows a similar dynamic in mice and pig PGC, with partial demethylation followed by remethylation after arrival to the genital ridges. The major changes in H3K9me2 and H3K27me3 occur in migratory PGC prior to their arrival to the genital ridge and are concurrent with the G2 arrest. The timelines for embryonic age are aligned according to the time points of PGC specification and arrival in the genital ridges for both species. Coloured boxes on the left hand side show the level of each epigenetic mark in somatic cells. Coloured lines depict presence of the indicated epigenetic marks at respective time points, and the lack of colour reflects the absence of the marks.

The present study establishes that pig migratory and gonadal PGC undergo an overall sequence of epigenetic reprogramming remarkably similar to that described in mice. First, gonadal PGC undergo extensive demethylation in the imprinted *IGF2-H19 *cluster. Secondly, the DMR2 of *IGF2R *is demethylated precociously in pre-gonadal PGC, specifically in male PGC. Thirdly, retrotransposable elements undergo progressive demethylation in PGC colonizing the primitive gonad. Finally, the changes in DNA methylation are preceded by reprogramming of H3K9me2 and H3K27me3 in migratory PGC. Although the period of time required for accomplishing these events is more than three times that required in mice (Figure 6), the dynamic reprogramming occurs at equivalent developmental stages as demonstrated in rodents, indicating that the difference probably stems from the fact that development is slower in the pig. Together these results support the idea that the epigenetic reprogramming of PGC is conserved in mammals. The extended time frame provides a useful window of opportunity for detailed dissection of the sequence of events leading to the reprogramming of PGC in slow developing embryos. For instance, the precocious demethylation observed for *IGF2R *in male pig PGC, highlights the advantage of having an extended window of time for studying these reprogramming events. Finally, a better understanding of the dynamic events during germ cell establishment may contribute to designing new strategies for the derivation of EG cells.

## Methods

### Embryos collection

All the procedures involving animals have been approved by the School of Biosciences Ethics Review Committee (University of Nottingham, UK). Embryos were collected from British Landrace sows or Yorkshire X Landrace gilts artificially inseminated or mated 15 (n = 1), 17 (n = 14), 18 (n = 13), 21 (n = 1), 22 (n = 15), 25 (n = 14), 29 (n = 4), 31 (n = 11) and 42 (n = 18) days prior to embryo collections. Embryos were recovered from the pregnant uteri within between 30 min and 2 hrs of slaughter.

### Immunohistochemistry

One embryo of each of the stages E15, E17, E21, E22, E25, E31 and E42 were fixed in 4% paraformaldehyde (PFA) in PBS overnight at 4°C. Tissue was hereafter dehydrated through increasing ethanol concentrations to xylene and embedded in paraffin. Transversal sections of 4-5 μm thickness containing the PGC were collected on SuperFrost Plus microscope slides (Menzel, Braunschweig, Germany).

### Tissue preparation for methylation analysis

Hindgut or genital ridges/early gonads were dissected from each embryo and roughly chopped before treatmentwith 0.1% collagenase/0.1% dispase for 11 minutes and subsequently 1 minute in 0.25% trypsin with EDTA at 37°C. Tissue was disintegrated by gentle pipetting after addition of Dulbecco's Modified Eagle Medium (DMEM) with 4-10% fetal bovine serum (FBS) and centrifuged 5 minutes at 600 × g. Cells were resuspended in FBS with 10% DMSO and stored in liquid nitrogen up to 10 weeks.

### Sequence homology

The putative *IGF2R *gene was identified by aligning the porcine partial coding sequence (Accession number AF339885) to the porcine genome (assembly version 8, Pre.Ensembl). The promoter region and exon 1 of the gene were deduced using the annotated *IGF2R *gene sequences of Bos Taurus (Accession number NM174352). The putative DMRs were identified by the freeware CpG Island Searcher [50].

### DNA extraction, gender determination and bisulfite conversion

Genomic DNA was extracted from porcine embryo tissue using Blood and Tissue DNA extraction kit (Qiagen, Hilden, Germany). The amount of extracted DNA was quantified on a NanoDrop spectrophotometer (Thermo Scientific, Waltham, MA, USA) and a maximum of 1 μg was used for bisulfite conversion. For gender determination we followed the protocol reported by [51]. Primers used are presented in Table 1. For bisulfite mutagenesis DNA was converted with EZ DNA Methylation-Gold kit (Zymo Research, Orange, CA, USA) and eluted in 10 μl nuclease free water following manufacturer's instructions.

### PCR amplification of bisulfite converted DNA

The bisulfite converted DNA was amplified by PCR. All primers, annealing temperatures and sizes of products are listed in Table 1. The PCR amplification consisted of a denaturing step of 5 min at 95°C followed by 50-52 cycles of 30 sec at 94°C, 30 sec at 57°C - 64°C and 1 min at 72°C. Finally, there was an extra elongation step of 15 min at 72°C. The amplified products were analysed by electrophoresis on 2% agarose gels. The amplified products were sequenced by direct sequencing after purification with Qiagen Gel Extraction kit (Qiagen, Hilden, Germany) or as individual clones after transformation using pGEM-T EasyVector System (Promega, Charbonniéres, France) in Escherichia coli DH5α. The obtained nucleotide sequences were analysed with the freeware Chromas Lite (Technelysium Pty Ltd). The methylation level of repeat sequences was calculated using the approach proposed by Yang et al. [52]. The method is based on the assumption that the mutation rate for CpG → TpG is identical on the two strands. Briefly, the number of potential CpGs in the investigated sequence was identified for all positions where one or more of the clones had a methylated CpG (See Table 1 for approximate numbers of investigated CpGs). Unmethylated CpGs were then calculated as TpGs deducted the number of TpAs (representing TpG mutations on the opposite strand) in the potential CpG positions. The efficiency of the genomic DNA conversion was evaluated by the number of non-converted non-CpG cytosines and no clones carrying more than one of these were included in the analyses.

### Immunohistochemistry on PFA fixed, and paraffin embedded tissue

Sections were deparaffinated in xylene and rehydrated through descending concentrations of ethanol. The epitopes were demasked by 15 minutes microwave boiling of the slides in TE-buffer (0,01 M Tris, 0,001 M EDTA), pH 8.0 (AppliChem) or 0.01 M citrate buffer (pH 6.0) followed by 15 minutes cool down and 15 minutes wash in demineralised water. Tissue was permeabilised in 1% Triton X-100, blocked in 2% BSA/PBS prior to 1 hour incubation with primary antibodies; rabbit monoclonal anti-H3K27me3 (Upstate; 1:200), mouse monoclonal anti-H3K9me2 (Abcam, 1:200) and goat polyclonal anti-OCT3/4 (SantaCruz; 1:200). Negative controls were incubated in blocking buffer. After extended washes, the sections were incubated for 40 minutes with secondary antibodies; Alexa Fluor ^® ^594 conjugated donkey anti-goat IgG (Invitrogen; 1:250), Alexa Fluor ^® ^488 conjugated donkey anti-rabbit IgG (Invitrogen; 1:250) and Alexa Fluor ^® ^488 conjugated donkey anti-mouse IgG (Invitrogen; 1:250). For chromogenic detection the ABC technique was performed using the Vectastain Elite ABC kit (Vector Laboratories, Peterborough, U.K.) with DAB (Vector Laboratories, Peterborough, U.K.) as a substrate to visualise the positive cells. The sections were counterstained with haematoxylin and mounted using DPX mounting media (VWR International Ltd., Poole, U.K.). For immunofluorescence slides were mounted in Fluorescence Mounting Medium (DakoCytomation) and pictures of areas containing PGC were captured in 40× magnification with Leica DMRB fluorescence microscope through Leica DFC350FX camera.

### Immunocytochemistry on ethanol fixed cell suspensions

Cell suspensions were thawed and added DMEM medium with 10% FBS. The cells were spun down and resuspended in medium twice to wash out DMSO before ice cold 99% ethanol was added dropwise to a final concentration of 70%. Cells were fixed at -20°C for 20 min. Before fixation, the suspension was filtered through a 30 μm nylon mesh (Miltenyi, Bergisch Gladbach, Germany) to ensure single cell suspension. Cells were washed twice in PBS with 0.1% Tween-20 and 1% BSA, permeabilised 30 min in 2% Triton X 100 with 0.1 mg/ml RNase A. The cells were resuspended in 5% BSA in PBS and incubated 1 hour 4°C to block unspecific antibody binding. Cells were incubated with goat anti-OCT3/4 antibody over night at 4°C (SantaCruz, 1:500 in blocking buffer), washed twice and incubated 1 hour RT with Phycoerythrin (PE)-conjugated donkey anti-goat IgG (AbCam, 1:100 in blocking buffer). Finally, the cells were washed three times before added 7-amino-actinomycin D (Invitrogen) to a final concentration of 4 μM. Cell suspensions were stored cold and in the dark until analysis. Negative controls were treated identically but incubated in blocking buffer instead of either the first or both antibodies. In addition, cells of the human embryonic kidney 293T cell line were used as negative cell samples while mouse embryonic stem cells were used as positive cell samples for adjustment of the flow cytometer.

### Fluorescence-activated cell sorting (FACS) analysis

Cell suspensions were analysed on an Altra Flow Cytometer (Beckman Coulter, Brea, CA, USA). Signals for forward scatter, side scatter and fluorescence (PE for OCT4 and 7-AAD for DNA content) were collected for a minimum of 50000 cells in each group. Representative FACS plots are shown in additional file 3. Data were analyzed using WinMDI (http://facs.scripps.edu/software.html; authored by Dr. J. Trotter (The Scripps Research Institute, California, USA), with FSC/SSC and pulse width gating to exclude doublets. Cells were sorted on the basis of their OCT4 expression into a negative and a positive sample. The positive samples contained a minimum of 500 putative PGC. Cell cycle analysis was carried out using the freeware Cylchred (Dr. T. Hoy, Cardiff University, School of Medicine (Cardiff, UK) to give the proportion of cells in each phase of the cell cycle.

## Authors' contributions

SMWH conceived and designed the study, performed experiments and wrote the paper. NC performed gendertyping and bisulphite sequencing analysis. DAC contributed with sample collection and immunocytochemistry. PDT participated in the design and coordination of the study. RA conceived, designed and coordinated the study, performed cloning experiments and wrote the paper. All authors read and approved the final manuscript.

## Supplementary Material

Additional file 1 **Germ cell cords in a male E42 pig gonad**. A section of a male gonad shows OCT4 staining (brown) in germ cells organized into testicular cords. Scale bar 20 μm. Click here for file

Additional file 2 **Representation of the IGF2R gene**. **A**. The exon/intron structure of the coding region is indicated by red bars and connecting lines, respectively. The coding sequence is positioned on the reverse strand of chromosome 1. The graph below shows the CG content of the sequence. Two CpG islands are identified (asterisk) in the promoter region and intron 2, respectively (Modified figure from http://www.ensembl.org). These positions correspond with CpG islands known from other species, and was used for the methylation analysis in the present study. **B**. shows the two islands identified on http://www.cpgislands.com each, with indication of the position of the bisulfite primers used (blue arrows). The position of exon 1 also is indicated. Click here for file

Additional file 3 **FACS plots of sorted PGC**. Porcine PGC were sorted on the basis of their specific OCT4 expression. Sorting was managed using the software WinMDI through manually determined gates for the different populations of cells. Representative plots from the sorting are shown for cell suspensions from embryos E22, E25 E29, E31 and E42. The square (R2) in the plot indicates the OCT4 positive gates. Plots show OCT4 staining intensity versus linear forward scatter. Click here for file
