# Epigenetic reprogramming in the porcine germ line

**DOI:** 10.1186/1471-213X-11-42

**Published:** 2011-06-28

**Authors:** Sara MW Hyldig, Nicola Croxall, David A Contreras, Preben D Thomsen, Ramiro Alberio

**Affiliations:** 1 Department of Basic Animal and Veterinary Sciences, Faculty of Life Sciences, University of Copenhagen, 1870 Frederiksberg C, Denmark; 2 Division of Animal Sciences, School of Biosciences, University of Nottingham, Loughborough, LE12 5RD, UK

## 

After the publication of this work [1] we became aware that Table one had inadvertently been omitted from the final version of the article. The table is now included in this document as Figure 1.

**Figure 1:**
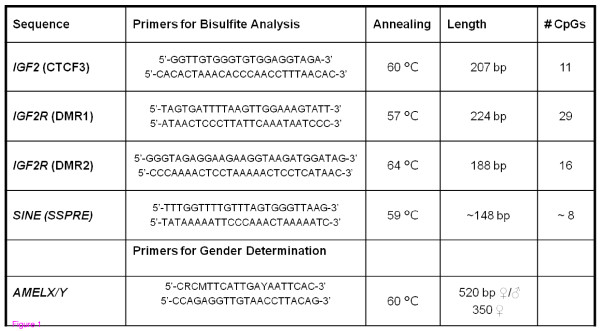
**Table one from Epigenetic reprogramming in the porcine germ line**.

We regret any inconvenience that this might have caused.

## References

[B1] Hyldig SM Croxall N Contreras DA Thomsen PD Alberio R Epigenetic reprogramming in the porcine germ line BMC Dev Biol 2011 11 11 10.1186/1471-213X-11-11 21352525 PMC3051914

