# Supplementary figures and images for: Epigenetic reprogramming in the porcine germ line
Source: BMC Dev Biol. 2011 Feb 25;11:11. doi: 10.1186/1471-213X-11-11 (PMC3051914; doi:10.1186/1471-213X-11-11)

**E42**

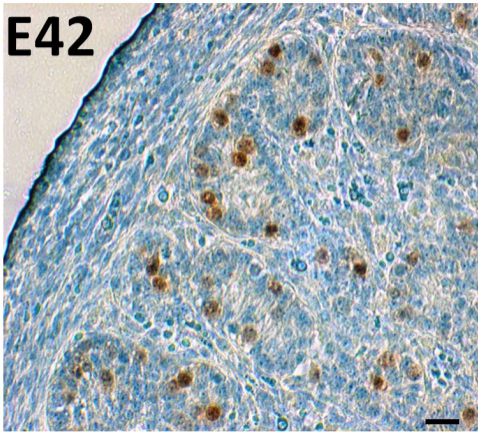

Supplement: Additional file 1 — Germ cell cords in a male E42 pig gonad. A section of a male gonad shows OCT4 staining (brown) in germ cells organized into testicular cords. Scale bar 20 μm. [file 1471-213X-11-11-S1.PDF]

A

*IGF2R* coding region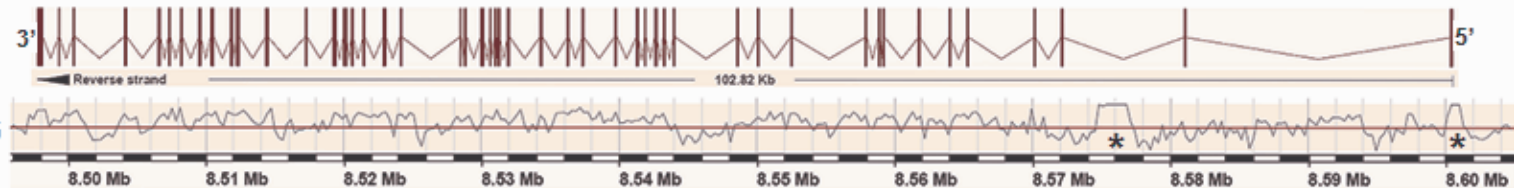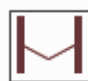

Exon-Intron-Exon

B

Predicted CpG islands

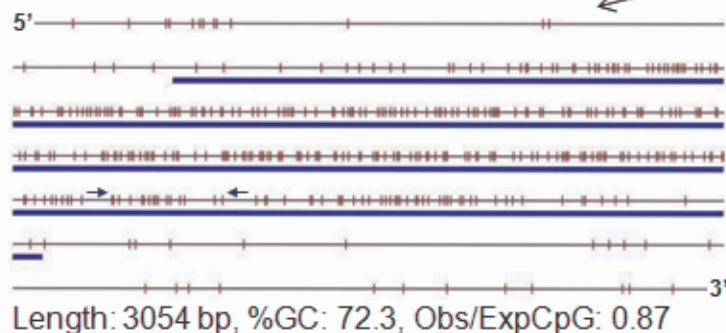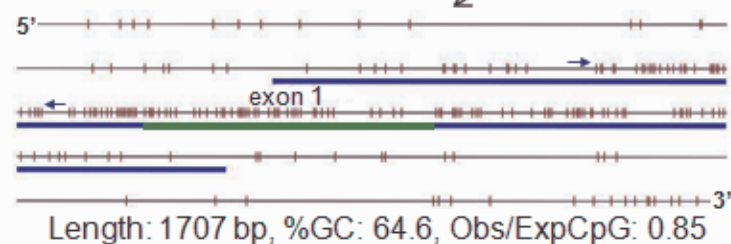

Supplement: Additional file 2 — Representation of the IGF2R gene. A. The exon/intron structure of the coding region is indicated by red bars and connecting lines, respectively. The coding sequence is positioned on the reverse strand of chromosome 1. The graph below shows the CG content of the sequence. Two CpG islands are identified (asterisk) in the promoter region and intron 2, respectively (Modified figure from http://www.ensembl.org). These positions correspond with CpG islands known from other species, and was used for the methylation analysis in the present study. B. shows the two islands identified on http://www.cpgislands.com each, with indication of the position of the bisulfite primers used (blue arrows). The position of exon 1 also is indicated. [file 1471-213X-11-11-S2.PDF]

Screen shots from  
WinMDI software

**E22**

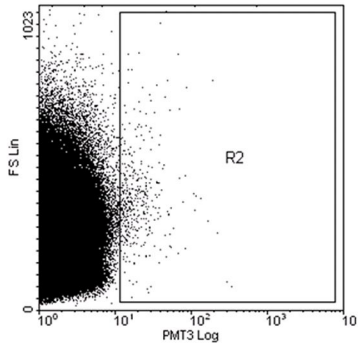

**E25**

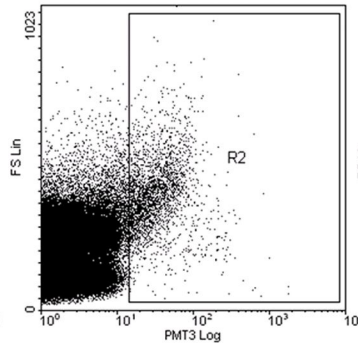

**E29**

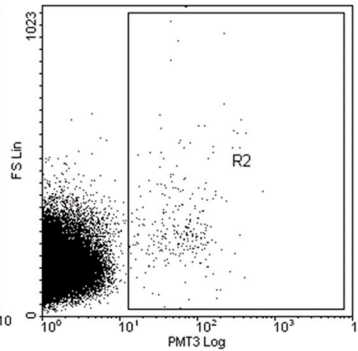

**E31**

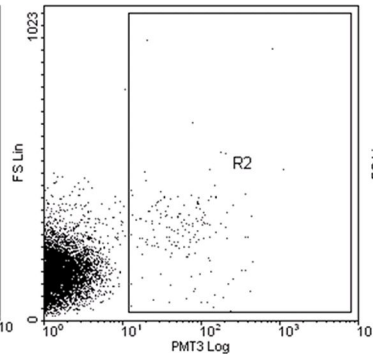

**E42**

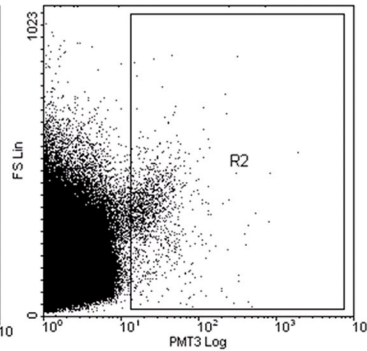

Supplement: Additional file 3 — FACS plots of sorted PGC. Porcine PGC were sorted on the basis of their specific OCT4 expression. Sorting was managed using the software WinMDI through manually determined gates for the different populations of cells. Representative plots from the sorting are shown for cell suspensions from embryos E22, E25 E29, E31 and E42. The square (R2) in the plot indicates the OCT4 positive gates. Plots show OCT4 staining intensity versus linear forward scatter. [file 1471-213X-11-11-S3.PDF]
